# 

**Published:** 1901-04

**Authors:** 


### INDEX TO VOLUME III.

### A

Accidents, Scientific Study of .......... 643
Acetanilid Poisoning .......... 864
Actinomycosis, A case of .......... 577
Albuminuria without Organic Renal Lesions .......... 217
Alcoholism Among Women .......... 774
Alum Enema after Abdominal Operations .......... 274
American Medical Association .......... 286
American Medical Association, Change in Constitution of .......... 352
American Proctological Society .......... 237
Anarchist and Substitutor, the .......... 496
Anemia .......... 522
Anesthesia .......... 600
Angina Pectoris .......... 667
Annual and Analytical Cyclopedia .......... 339
Antacrida Tincture .......... 327
Anterior Anethritis, Treatment of .......... 488
Anthrax, A case of .......... 377
Antiseptic and Eliminative Treatment of Typhoid Fever .......... 365
Antivivisectatonists Again, The .......... 617
Apropos of Buttons .......... 344
Army Canteen, The .......... 255
Aseptic Adhesive Strips in Closure of Wounds .......... 765
Assassination of the President .......... 450
Atlanta Society of Medicine .......... 830
Atlas and Epitome of Labor and Obstetrics .......... 189
Atlas and Epitome of Nervous System and Its Diseases .......... 189
Atlas and Epitome of Obstetric Diagnosis and Treatment .......... 190
Atlas and Epitome of Ophthalmology .......... 187
Attempts on the Lives of Previous Presidents .......... 473
Automatic Safety Valve Stopper .......... 284

### B

Baneful Effects and Curability of Catarrhal Inflammation of the Upper Air Passages .......... 161
Bladder Cases, Some Obstinate .......... 791
Bovine and Human Tuberculosis .......... 387
Brief Manual of Prescription Writing .......... 754
Burdette's Oration .......... 411

C

Cæsarean Section, Self-Performed ..... 432
Cancer, Treatment of External..... 79
Carbolic Acid Gangrene..... 158
Carbolic Acid and Tansy Poisoning..... 607
Catching Cold..... 709
Chancre, The Unrecognized..... 862
Charlatanism in Cure of Alcohol..... 50
Cheap John Syphilographers..... 501
Chemical Experiences with Adrenalin..... 198
Chemistry, Manual of (Simon)..... 561
Child with Shingle Nail in Windpipe..... 596
Children, Diseases of..... 42
Choroid, Rupture of..... 531
Christian Science, A Facer for..... 131
Christian Science and Consumption..... 206
Clinical Pathology of the Blood..... 397
Clinical Report of Pepto-Mangan..... 576
Clinical Society of Maryland..... 16
Cocaine Habit, The..... 859
Colic and Colds in Infants..... 43
Collective Investigation Nitrate of Silver in Tuberculosis..... 386
Compend of Physiology..... 143
Consumption in Relation to General Public..... 793
Consumption, Suppression of..... 649
Contagious Diseases of Childhood..... 188
Continued Fever in South Carolina..... 443
Continued Malarial Fever..... 526
Coroner's Inquest..... 424
Corporal Punishment..... 661
Coughs..... 778
Crusade Against Tuberculosis..... 329

D

Dangers from Milk..... 283
Deafness, Study of..... 73
Diagnosis of Abdominal Diseases..... 65
Dictionary, Medical (Dorland)..... 628
Difficulties in Management of Social Evil..... 721
Disease Odors..... 499
Disease of Philistines..... 128
Diseases of Women, Hints on Pelvic..... 327
Dysenteric Diarrhea, Treatment of..... 224
Dyspeptics, Rules for..... 213

E

Eczema (Bulkley)..... 338
Endometritis, Treatment of..... 779

Epidemics of Smallpox in Ancient Times .... 68
Epistaxis .... 360
Epoch Making in Medicine, Recent .... 656
Esophagus, Stricture of .... 793
Etiology of Constipation .... 631
Excision in Tuberculosis of Joints .... 145
Extinction of Gynecology .... 773
Extra-Uterine Pregnancy .... 1
Eye, Foreign Bodies in .... 485
Eye, Treatment of by General Practitioners .... 637

F

Failure of Knife in Treatment of Cancer .... 757
Fallibility of Scientific Evidence in Medical Jurisprudence, The .... 534
Fatalities of Spinal Cocainization .... 214
Favorite Prescriptions .... 339
Fee Splitters, The .... 864
Flies .... 251
Forceps in Childbirth, Use of .... 215
Foreign Body in Vagina .... 539
Fractures of Humerus, Suggestions on Treatment of .... 478
Fractures, The Treatment of (Scudder) .... 39

G

Genito-Urinary Organs .... 55
Georgia State Sanitarium .... 648
Gonorrhea as a Social Problem .... 217
Gonorrhea, Treatment of .... 216
Gray's Anatomy .... 695
Growing Pains .... 348

H

Handsome Group of Portraits, A .... 720
Hare's System of Therapeutics .... 190
Health Officer for Atlanta, A .... 29
Hemorrhage, to Control in Operations on Heartbeat .... 782
Hemorrhoids, Treatment of .... 360
Hereditary and Acquired Characteristics of Social Questions .... 512
Hints on Care of Infants .... 179
Historical Notes of McKinley's Case .... 482
Holt, Dr. W. F .... 544
Hospitals of Japan, The .... 191
Hydrophobia, three cases of .... 696
Hygiene, Principles of (Bergey) .... 559
Hyperacidity as a Cause of Skin Disease .... 212
Hypnotism (DeLaurence) .... 42
Hysterical Religion and Science .... 493

### I

Impotence, Sexual (Vecki) .......... 559
Increasing Sterility of American Women .......... 416
Inebriety, Data Relative to .......... 761
Infant Feeding .......... 85
Infections in Diseases of Women, The Rate of .......... 263
Insanity in Women from Gynecologic Standpoint .......... 774
International Clinics .......... 42
"Is Gonorrhea Curable?" .......... 647

### K

Kidney, Medical Treatment in Diseases of the .......... 468
Kidneys, Diuretics in Relation to, Condition of .......... 789

### L

Laryngeal Phthisis (Lake) .......... 140
Lea's Series of Pocket Text-Books .......... 753
Legislation in Prevention of Crime .......... 505
Leprosy in the United States .......... 288
Libertinism and Marriage .......... 695
Local Anesthesia in Hemorrhoidal Operations, etc .......... 63
Long, Dr. Crawford W ..........
Lupus Treated with Oils, Case of .......... 383

### M

Macon Medical Society .......... 685
Madstone, The .......... 181
Marriage of Criminals .......... 546
Mastoid Disease .......... 590
Mastoiditis, Report of Cases of .......... 656
Materia Medica (Shoemaker) .......... 39
Medical Association of Georgia .......... 112
Medical Directory, Standard .......... 844
Medical News Formulary .......... 752, 142
"Medicine in the Gutter" .......... 180
"Memoria in Eterna" .......... 648
Methylen Blue in Methritis .......... 863
Mucous Disease .......... 359

### N

Nasal Catarrh, Treatment of .......... 718
Nasal Obstructions (Shield) .......... 142
Nasal Sarcoma .......... 675
Nasal Sprays .......... 433
Naso-Pharyngeal Syphilis .......... 603
Negro Traits .......... 741

Nervous and Mental Diseases (Bershing) .... 629
Neurasthenia, Sexual .... 70
New Nose Cup, A .... 432
Non-Surgical Treatment of Pelvic Exudases .... 317
Notes on Augusta Meeting .... 133
Notice .... 330
Notice to Subscribers .... 72
Nose and Throat (Coakley) .... 338
Nursing (Hampton) .... 140

O

Ophthalmic Diagnosis (Haab) .... 187
Objects of the Organization .... 726
Observations, a Few .... 744
Organization .... 754
Our New Problem .... 816
Outlines of Physiology (Jones) .... 695
Overworked School Children .... 861

P

Parson and His Blue Ball .... 824
Peculiar Remedies Among Boers .... 60
Physical Diagnosis (Tyson) .... 753
Physician's Pocket Account Book .... 630
Physiologic Therapeutics (Lotis-Cohen) .... 262
Physiologic Therapeutics of Religious Mania .... 304
Poisons, Memoranda of (Tanner, Leppman) .... 143
Practical First Principles (Leuf) .... 455
Practical Medicine (Nothnagel's) .... 627
Practical Medicine Series .... 844
Practical Medicine Series, Vol. 2. .... 696
Practical Therapeutics (Hare) .... 37
Practice of Medicine (Eichborst) .... 187
Practice of Medicine as a Source of Income .... 201
Preliminary Anesthenia as an Aid to Diagnosis of Fracture .... 167
Prevention of Ophthalmia Neonatorum .... 785
Principles of Surgery (Senn) .... 262
Prize Medals (Senn) .... 576
Progressive Medicine .... 560
Progressive Medicine, Vol. I .... 42
Prolapsus Recti in Children .... 786
Prostitution and Venereal Disease .... 768
Psychology of Sex (Ellis) .... 752
Puerperal Infection .... 671
Puerperal Sepsis .... 420
Pulmonary Consumption (Mays) .... 41

### Q

Questions Asked by State Examining Board ........ 135
Quiet Effusion Into the Knee-Joints ........ 355

### R

Rape in the South, Increase of ........ 497
Rational Treatment of Gout ........ 787
Rehabilitation of Medical Expert Witness ........ 170
Remedy Proposed for Evil of Substitution ........ 720
Report of Sociological Committee ........ 540
Rheumatism, Specific for Chronic ........ 783
Richmond Academy of Medicine ........ 324, 682 826
Roentgen Society ........ 504

### S

Saline Transfusion ........ 11
Scarlatinal Nephritis ........ 853
Scientific Slang ........ 834
Scientific Study of Criminal, etc ........ 575
Septic Infection ........ 357
Sexual Therapeutics, Success in ........ 280
Skin Diseases (Jackson) ........ 396
Smallpox ........ 845
Society, Parturition and Posterity ........ 733
Spina-Bifida, Report of Cases ........ 842
Splenectomy, a Case of ........ 289
Stomach Contents, Examination of ........ 801
Stomach, Diseases of the ........ 38
Subphrenic Abscess ........ 98
Sugar in Urine, Surgery in ........ 788
Suit of Damages ........ 388
Suppression of Consumption ........ 649
Surgeons of Confederate Army and Navy, The ........ 771
Surgery by American Authors (Park) ........ 630
Surgery During the War ........ 491
Surgery, Twentieth Century ........ 562
Surgical Case, Report of a ........ 449
Surgical Cases, Some ........ 398
Syphilis and Tabes, Relation of ........ 687
Syphilis, Factors Influencing Severity of ........ 438
Syphilis, Treatment of ........ 715

### T

Taxemia of Pregnancy ........ 215
Tonsillitis from Standpoint of General Practice ........ 312
Tracheotomy, Unique Complication of ........ 322

Tuberculosis, Origin and Dissemination .......... 870
Typhoid Fever in Country Practice .......... 373
Typographical Errors .......... 780

**U**

Ulcers, Diagnosis of .......... 381
Uterine Fibromata (Stanmore) .......... 396

**V**

Vaccination .......... 296
Vaccination, Tetanus from .......... 784
Venereal Diseases (Sturgis & Cabot) .......... 42
Virchow Fund .......... 126

**W**

When to Watch Out .......... 251
Whooping Cough .......... 785
Why Does an Acute Abscess Get Well .......... 216

**X**

X-Ray in Surgical Cases .......... 361

**Y**

Yellow Jessamine, Tincture of .......... 221

### AUTHORS.

Brawner, James N ... 807
Bankston, R. C ... 649
Boynton, C. E ... 607
Bryson, J. P ... 289
Bullard, W. L ... 596
Campbell, J. L ... 11
Carson, M. F ... 361
Childs & Champion ... 217
Conway, W. B ... 522
Cox, R, P ... 73
Crawford, J. M ... 656
Deas, W. A ... 228
Downey, C. W ... 539
Ehrenfest, H ... 317
Ellett, E. C ... 322
Ferguson, E. G ... 741
Garner, J. R ... 812
Gaston, J. McF. Jr ... 98
Gray, A. L ... 312
Greenleaf, J. S ... 449
Grindon, Jos ... 296
Haymore, G. P ... 600
Hiers, J. L ... 793
Hobbs, A. G ... 161
Hoover, F. P ... 590
Hopkins, E. G ... 801
Hopkins, T. S ... 534
Howard, N. F ... 383, 221
Howell, J. R. G ... 526
Hubbard, T. V ... 365
Hudson, W. H ... 577, 158
Hutchins, M. B ... 79
Keeble, J. B ... 505
Kime, R. R ... 726, 522
Knight, J. A ... 373
Lindorme, C. A. F ... 504
Manley, T. H ... 167, 381
Moore, J. H ... 443
Nuckols, N. E ... 667
Parks, W. B ... 661
Ravold, A ... 377
Robins, C. R ... 1
Roy, Dunbar ... 675, 433, 531
Smith, F. S. K ... 816
Smith, W. Monroe ... 671
Tetau, J ... 679
Thompson, R. M ... 733
Tovian, Geo ... 224
Visanska, S. A ... 85
White, G. R ... 721
Williams, H. J ... 145
Wolff, Bernard ... 438

This Journal and Atlanta Semi-weekly Journal one year for $1.00 cash in advance. No checks. Strictly to new subscribers.

**This Journal and Atlanta Semi-weekly Journal one year for $1.00 cash in advance. No checks. Strictly to new subscribers.**

### NOTICE TO SUBSCRIBERS.

**We desire to explain that no offense was meant by our placing subscription accounts for collection. It was simply a matter of convenience. We cannot give the accounts as much attention as an agency can, hence are compelled to employ the agency. We only desire money justly due; never have, and never will knowingly accept subscription money not justly ours.**

